# Chemical Activation of Lignocellulosic Precursors and Residues: What Else to Consider?

**DOI:** 10.3390/molecules27051630

**Published:** 2022-03-01

**Authors:** Juan Alcañiz-Monge, María del Carmen Román-Martínez, María Ángeles Lillo-Ródenas

**Affiliations:** MCMA Group, Department of Inorganic Chemistry and Materials Institute (IUMA), Faculty of Sciences, University of Alicante, Ap. 99, E-03080 Alicante, Spain; jalcaniz@ua.es (J.A.-M.); mcroman@ua.es (M.d.C.R.-M.)

**Keywords:** chemical activation, biomass, lignocellulosic, activated carbons

## Abstract

This paper provides the basis for understanding the preparation and properties of an old, but advanced material: activated carbon. The activated carbons discussed herein are obtained from “green” precursors: biomass residues. Accordingly, the present study starts analyzing the components of biomass residues, such as cellulose, hemicellulose, and lignin, and the features that make them suitable raw materials for preparing activated carbons. The physicochemical transformations of these components during their heat treatment that lead to the development of a carbonized material, a biochar, are also considered. The influence of the chemical activation experimental conditions on the yield and porosity development of the final activated carbons are revised as well, and compared with those for physical activation, highlighting the physicochemical interactions between the activating agents and the lignocellulosic components. This review incorporates a comprehensive discussion about the surface chemistry that can be developed as a result of chemical activation and compiles some results related to the mechanical properties and conformation of activated carbons, scarcely analyzed in most published papers. Finally, economic, and environmental issues involved in the large-scale preparation of activated carbons by chemical activation of lignocellulosic precursors are commented on as well.

## 1. Introduction

Since the last century, exorbitant growth in all aspects of human activity has taken place, in part due to the rise of the human population itself. Thus, the world’s population has boosted from 2.5 billion in 1950 to tripling up to 8 billion in 2021, with the projections for 2050 of about 9.6 billion people [1]. The population rise will entail a great demand in food production and water purification, together with an increase in industrial manufacturing, all associated with large energy requirements. This notable increment in the activity of all sectors (industrial, agricultural, energy, and services) may lead to a greater increase in environmental problems. Among these serious concerns, having potable water both for agriculture and livestock, as well as for human consumption, is amongst the most important ones [2]. The United Nations considers it a priority objective, as stated in the Millennium Development Goals [2]. Within this context of air and water pollution control and water purification, attention is paid to the useful technologies to solve these problems, being adsorption on porous solids one of the best techniques for pollutants’ removal [3]. Activated carbons (ACs) are amongst the most widely used [4]. This is related to their excellent properties: variable (and high) specific surface areas, tunable porosities (from the point of view of pore volumes, pore sizes, and pore size distributions), tunable surface chemistry (surface functional groups of different nature, variable content, and proportions), high mechanical resistance, and good electrical conductivity. Thus, their consumption has increased in terms of quantity, given that they are used, among many other fields, for water purification [5]. The US Environmental Protection Agency recommends adsorption by activated carbons as the most appropriate technology for the elimination of organic pollutants present in aqueous media [5]. Moreover, the legislation by the Environment Protection Agency (EPA) in the US that regulates the control of acid and heavy metal emissions (mainly from thermal power plants) changed in 2011, and also a new treaty by the EPA Mercury and Air Toxics Standards (MATS) for the control of mercury emissions was applied in October 2013, which emphasizes the need of efficient adsorbents for environmental problems remediation. As a consequence of these interesting properties many applications related to adsorption, catalysis, and electrochemistry fields, among others, involve ACs.

In view of all this, it is not surprising that the demand for AC materials will increase in the coming years, and also their price, in part as a consequence of the increasing use of ACs in water treatment. As an example, the world’s production of ACs was 1.7 million tons in 2020, and the predictions for the next five years point to an annual increase of 3%, standing at about 2 million tons [6]. Thus, the activated carbons market is expected to reach USD 8.49 billion in 2025, at a compound annual growth rate of 11.4% [7].

ACs have been prepared since pre-Egyptian times, although it was during the 19th century that numerous trials were carried out to improve ACs properties and performance. These studies were collected by Ostrejko (1900), who published the patents that later derived the two most widely used methods for the production of ACs at an industrial level: the so-called physical activation and chemical activation, British Patent 14,224, 1900; French Patent 304,867, 1901; German Patent 136,792, 1901, and US Patent 739,104, 1903 [8]. Among these methods, since the 1980–1990s, there is an expanding interest in chemical activation, the topic of this research.

Chemical activation is (wrongly) generally defined as a solid–solid reaction that involves intimate mixing of a carbonaceous precursor with a chemical (activating) compound (traditionally in the form of a concentrated aqueous solution), followed by heat treatment [3,4,9,10,11,12]. It can be performed either in a single heating stage or in two stages, depending on the type of precursor [9,10,11,12,13]. In the case of carbon-rich precursors, such as coals and coke, the process is usually carried out in a single heat treatment step [9,10,14,15]. In the case of lignocellulosic materials, a pyrolysis step is generally carried out as the first heating stage to obtain a (bio)char, which is subsequently used as a precursor, being then mixed with the chemical compound solution, dried, and activated in a heat treatment [11,13,15,16,17,18,19]. In some cases, such as activation with hydroxides, the activating agent is preferably mixed in solid form to avoid its carbonatation, and also for economical and operative reasons [10,14,15,20]. Some studies devoted to hydroxides activation highlight that, in fact, chemical activation occurs via solid–liquid reaction because the activating agent usually melts before/during the activation procedure [11,21,22].

In general terms, the published papers on the preparation of activated carbons by chemical activation have focused on different activating agents, different precursors, and different (activation) experimental conditions, which have led to a wide variety of porosities. In most cases, the achieved porosity has been the main concern, together with the final yield, whereas surface chemistry has been less studied.

In parallel with studies devoted to the preparation of ACs, the use of these sorbents in a wide range of applications has been deeply investigated. Moreover, since the 2000s, the mechanism of chemical activation has also been analyzed with the aid of different experimental techniques [20,21,23,24], being the conclusions of those studies of great relevance for the efficient preparation of activated carbons.

Recently, all the previous knowledge/background about chemical activation has been applied to the use of lignocellulosic sources as precursors [11,12,13,18,19,25,26], in line with green chemistry principles. Thus, the development of efficient methods for preparing ACs from inexpensive and abundant lignocellulosic (biomass) residues from agricultural activities stands out [15,18,27]. As a result of the interesting properties for ACs chemically derived from lignocellulosic materials and residues, their use in a wide variety of applications, including energy storage or CO_2_ removal as examples, has received great attention [26,28,29,30,31,32,33,34]. However, despite the highlighted interest in chemical activation, a literature survey on the topic emphasizes that some important aspects have been scarcely investigated and merit a deep analysis.

On one hand, little attention has been paid to the physicochemical transformations of the lignocellulosic biomass components during heat treatment. Also, the obtained yields and porosity developments require some revision, comparing them with those obtained by physical activation of similar precursors. Moreover, although the importance of the porosity development is undoubtful, little attention has been paid to the characterization of other features, which determine the use of these adsorbents in novel and important applications. For example, only a few studies have analyzed the surface chemistry of the activated carbons, and no clear conclusions have been extracted about the relationship between activation conditions and surface chemistry. Other features, such as density, granulometry-morphology, mechanical properties, electrical properties, or the presence of heteroatoms have been less analyzed. Very little attention has been paid, as well, to scaling up laboratory procedures or to the main economic and environmental issues and concerns, such as the possibility of recycling the activating agents for further activations. Therefore, this work aims to revise and comment on the published information devoted to some of those topics.

## 2. Preparation of Activated Carbons

Activated carbon is identified by the ACS registry number 7440-44-0 and from the point of view of chemical composition is mainly based on carbon (C). AC is macroscopically considered an amorphous structure derived from an allotropic modification of graphite; all forms of synthesized ACs contain heteroatoms (mainly O, H, N, P, and S) and, structurally, it can contain some crystalline domains and have different order degrees. The presence of heteroatoms is largely linked to the organic origin of each precursor and the activation process.

As mentioned, the ACs′ total porosity, pore size distribution and the nature/content of their surface chemistry can be “easily” tailored [10,18,25] by the appropriate selection of the precursor and the experimental activation parameters [10,11,13,18].

### 2.1. Precursors

One of the first advantages of chemical activation is its versatility in terms of the raw materials used as precursors for the production of ACs. Thus, any material and/or residue with a certain carbonaceous matter content can be used for the production of ACs [3,9,13,25,35], although its availability and price must be also evaluated. ACs can be prepared from coals, wood, lignocellulosic wastes, domestic wastes (paper, cardboard, plastics), industrial wastes (from oil industries, carbochemical synthesis, cellulose …), tires, or sewage sludge wastes (see Table 1, which compiles the raw materials that can be selected for the preparation of activated carbons). The final quality of the AC largely depends on the type of precursor, being the final application another aspect to consider when choosing it. Thus, in principle, precursors with high carbon and low ash (inorganic matter) content are the most suitable candidates [13,25], although other compositional features that will be discussed in more detail next, such as the lignin content in the lignocellulosic precursor, have an important influence as well [36].

Thus, because of the high carbon content in coals (see Table 2), they have been among the preferred options for getting developed porous structures (especially lignite, subbituminous, and anthracite), allowing the use of economic preparation procedures. These precursors, with a carbon content around 65–95%, have a developed micrographitic structure (depending on the maturity or range) and, so, their activation is relatively easy. However, these are not cheap raw materials, and they are located in certain parts of the planet. Therefore, traditional preparation and processing plants of ACs have been developed in areas close to the mines-deposits. This justifies that a target, for economic and environmental reasons, of replacing coal with (biomass) wastes, and understanding and optimizing the preparation procedures.

Data in Table 2 shows the high carbon contents in coals, but the corresponding values in biomass residues (see Table 3) made them outstanding precursors as well. In the latter case, the use of diverse plant parts (including core, stems, shells, peels, flowers, fruits, seeds, stones, husks, leaves, fibers, grass, starch, among others), is an important advantage [25]. Despite the huge variety of lignocellulosic materials, most of them present a high carbon and oxygen content, around 40–60% and 40–45%, respectively. Note that whereas their carbon content is lower than for most coal precursors, their oxygen content is larger (even around double in some cases). From an environmental point of view, the lower sulfur content for biomass-origin raw materials in comparison with coal precursors must also be remarked. However, no micrographitic structure is developed in biomass materials and, for this reason, they are usually submitted to a thermal process before being used as activation precursors after which some increase in their carbon content occurs, concomitant to a slight porosity development (in some cases).

Thus, the preparation of activated carbons stands out for the wide variety of procedures used. Once the precursor is known/selected, three main preparation variables can be highlighted (summarized in Figure 1):

- The activating agent. It covers H_2_SO_4_, ZnCl_2_, H_3_PO_4_, KOH, and NaOH, etc., showing from neutral to acid or basic character. It reacts in a controlled manner with carbon, consuming some of it, and creating porosity [4,9,11,21,25]. Literature states that, as a general trend, ZnCl_2_ and H_3_PO_4_ are the most suitable activating agents for low-ordered precursors (including lignocellulosic precursors and low-rank coals), whereas alkaline hydroxides are more suitable for the activation of highly ordered ones (i.e., high-rank coals, such as anthracites or carbon nanotubes) [10,39].
- Heating treatment. Either pyrolysis followed by activation (if two-step chemical activation-procedure is used) and/or a single step chemical activation are required for preparing activated carbons, being the heating rate, the activation temperature, and the holding time at the activation temperature among the most influencing experimental parameters [25,37]. In any case, conventional furnaces based on standard electrical resistances are commonly used, and the heat transfer occurs from the gas to the sample to be activated, either in slow or in flash conditions [9,40]. In conventional furnaces, heat is transferred from the external surface to the internal part of the sample to be activated. In other cases, the heat transfer can also occur via microwave heating [19,25]; heat is transferred from the internal parts of the particles (i.e., lignocellulosic and activating agent) towards their surface. More recently, hydrothermal treatments have been implemented for chemical activation, either for the carbonization (pyrolysis) step or for the direct activation [41,42,43,44]. In these conditions, water media (containing the activating agent dissolved if activation) acts as a “reactant”, favoring and accelerating the desired processes [41].
- Atmosphere. The heating occurs in presence of a controlled gaseous atmosphere, commonly flowing gas [11,25], which removes the evolved gaseous products (favoring the activation reaction [21]) and facilities the heat transmission to the solid sample. Different atmospheres, mainly nitrogen and steam, can be used [12,13,18,19,25,37,45], being the flow rate used an important parameter influencing the process.

**Table 3:** Elemental analysis, lignocellulosic composition, and ash content of biomass precursors weight percentage on air-dried basis [12,18,19,37,46].

Precursor	Ultimate Analysis (wt %)				Lignocellulosic Composition (wt %)			Ash (wt %)
	C	H	O	N	Cellulose	Hemicellulose	Lignin	
Almond shell	49.5	6.3	44.0	0.2	32	26	25	2.2
Coconut shell	48.7	6.3	43.4	1.5	41	27	29	4.0
Palm shell	47.8	6.0	45.3	0.9	30	17	53	4.2
Hazelnut shell	47.0	6.5	46.0	1.0	25	28	42	1.4
Peanut shell	41.5	5.6	2.2	2.1	45	8	33	4.3
Palm kernel shell	43.6	4.9	51.6	0.5	30	21	47	2.4
Peach stone	45.9	6.1	47.4	0.6	46	14	33	1.5
Olive stone	45.0	5.8	48.3	0.2	32	33	30	2.1
Date pits	45.6	7.1	46.5	0.7	24	27	22	1.0
Orange peel	46.6	6.1	47.1	0.2	65	5	20	1.0
Tomato waste	59.0	8.2	29.8	0.3	33	24	35	1.6
Tobaco stalk	46.2	6.1	43.4	2.4	42	28	27	2.4
Cotton stalk	41.2	5.0	34.0	2.6	39	17	29	5.0
Corn stalk	45.5	6.2	41.1	0.8	23	43	16	7.5
Corn cob	46.3	5.6	42.2	0.6	43	37	15	3.5
Olive tree pruning	49.9	6.0	43.4	0.7	29	21	27	5.0
Vineyward pruning	47.6	5.6	41.1	1.8	38	34	27	3.5
Peach tree pruning	53.0	5.9	39.1	0.3	31	28	28	3.7
Oats straw	46.0	5.9	43.5	1.1	35	37	18	8.7
Sunflower straw	52.9	6.6	35.9	1.4	32	19	22	9.0
Barley straw	46.2	5.8	41.9	0.6	38	35	16	7.0
Rice straw	49.5	6.1	44.3	0.2	38	32	12	20.0
Wheat straw	42.7	5.6	39.7	0.3	33	20	15	3.7

In some cases, prior to starting the activation, some pre-treatments over the lignocellulosic material are required, including size reduction, screening to desirable particle size, washing, and drying [12]. The washing step of the raw precursor is particularly important to eliminate any contaminant or soil that could influence the precursor’s quality. It may involve acid washing to eliminate soluble and insoluble metals, ash, and lignin; hot water washing soluble fractions, or alkaline washing, to react with silicon present in some biomass materials [12]. 

### 2.2. Processes: Pyrolysis and Activation

As already indicated, two heat treatment procedures are common when using lignocellulosic residues as precursors for chemical activation [11,13,15,16,17,18,19,25], in contrast to a unique one usually employed for coal precursors [9,10,14,15]. A (first) heat treatment is required due to the “low” carbon content in these precursors and the absence of a developed microcrystalline structure within them. This step can lead to a slight porosity development. This fact was already known in ancient times since these precursors only exhibited adsorbent properties after pyrolysis (carbonization). After the carbonization step, the “system” precursor-activating agent is submitted to a second heat treatment step, called activation, being this stage responsible for the porosity generation [25,37]. In some cases, just one carbonization-activation heat treatment step can be used [4,9,15,25,37], and some studies have compared the porosity achieved in one-step activation with that developed by consecutive carbonization and activation treatments [15]. Pyrolysis and activation are explained next in more detail.

#### 2.2.1. Carbonization or Pyrolysis

To start this section, it is interesting to clarify the differences between two terms that are usually indistinctly used, pyrolysis and carbonization. *Pyrolysis* is a general term for thermal decomposition or chemical change in an inert atmosphere through heating [47]. *Carbonization* is a slow heat-treatment process, in which the primary goal is the formation of carbon materials from organic matter [48]. Considering the aim of this study, the terms pyrolysis and carbonization can be indistinctly used since the thermal treatments that will be mentioned are applied in an inert atmosphere, at a low heating rate and with the purpose of obtaining a material with higher carbon content.

When dealing with physical activation, or with chemical activation of some precursors and/or activating agents, a first carbonization step is applied, consisting of thermal treatment in an inert atmosphere (without the addition of solid or liquid chemical substances). The objective is to obtain a higher final carbon yield. Thus, temperatures between 400–900 °C can be used, although they are usually higher than 600 °C so that aromatization and condensation take place [4,9], although, with some precursors and/or conditions, the range of carbonization temperatures may be different (i.e., for hydrothermal carbonization temperature normally ranges 200–350 °C).

As a result of the temperature increase, some heteroatoms (other than carbon) are eliminated in the form of gaseous compounds. The exit of these gases leaves unsaturated parts of the molecules (radicals), which thus facilitate radical addition reactions [49], favoring bonds between the aromatic rings from the different molecules present in the precursors. In lignocellulosic materials, the pyrolytic breaking of the bonds between carbon and the different heteroatoms (mainly hydrogen and oxygen and also, sulfur, nitrogen, and others) takes place at 170–650 °C [50]. Depending on their volatility, they are permanently eliminated as gases (CO, CO_2_, CH_4_, H_2_, H_2_O, …) or tar (high molecular weight species, which are volatile at carbonization temperatures, but condense at lower temperatures), leaving a solid residue with a high carbon content, called carbonized solid or (bio)char [11,17,18,25]. Depending on the final temperature reached, the biochar can contain up to 80–95 wt.% carbon [11,17,18,25]. Thus, after pyrolytic decomposition, three products are obtained: carbonized material, also called char (15–20 wt.%), tar (60–65 wt.%), and gases (20–25 wt.%) [13,17].

When preparing ACs, one of the objectives of the carbonization stage is to maximize the char (carbonized) fraction. For that purpose, it is necessary to know which are the most appropriate features of the precursor so as to obtain high carbonization yields. The analysis of the pyrolysis process in lignocellulosic materials is extremely complex as a consequence of the chemical and structural complexity of the organic macromolecules that constitute them [51,52,53]. These macromolecules can be classified into three large groups: hemicellulose, cellulose, and lignin (see mean contents in the different precursors, Table 3). Given the different chemical structures of hemicellulose, cellulose, and lignin, their thermochemical behavior is also different, and so are the numerous chemical reactions occurring in different temperature ranges (dehydration, depolymerization, bond breakage, formation of new bonds) [11,18,54]. Thus, hemicellulose is the least thermally stable, rapidly degrading between 200–350 °C [19,55]. Cellulose has greater stability, but it also degrades rapidly in the temperature range 300–400 °C [19,54]. In contrast, lignin decomposes slowly in a wide range of temperatures, 200–500 °C, being the component that contributes the most to char yield [19,56]. Therefore, from the point of view of charring/carbonization, residues with high lignin content are, in principle, suitable precursors to obtain ACs [12,13,25,37].

Parallel to the release of tar and volatile matter, a condensation process takes place due to bonding of unsaturated carbon atoms during the pyrolysis process [11,57]. The formation of aromatic sheets and the growth of these lamellae, which is correlated with the increase of the La (size of graphite lamellae) peak determined by XRD, is important from 600 °C [25,58]. These generated sheets, if the geometric arrangement during their growth allows it, will be stacked generating microcrystals. This process begins at 800 °C and, in turn, microcrystals are joined with their neighbors through interlocking links in a disorganized form, which gives rise to a poorly packed structure with free spaces that constitutes the pores in the char [57,58,59]. However, these spaces are generally filled (or their entry is partially blocked) by deposition of products emitted during decomposition, mainly tar, resulting in relatively low adsorption capacity chars [3,9]. Thus, pyrolysis/carbonization is not efficient for achieving a high porosity, and a subsequent activation stage is required to first eliminate these tar deposits and then widen the existing porosity, which increases the adsorption capacity of the then-called activated carbons [9].

#### 2.2.2. Chemical Activation

As explained, an activation process is required to get a noticeable porosity and, for that purpose, the use of a chemical/activating agent is required. The most common activating agents are H_2_SO_4_, ZnCl_2_, H_3_PO_4_, KOH, and NaOH [9,10,11,22,25]. In general, the literature states that the activating agents act both as dehydrators and oxidants. Thus, regarding the use of H_3_PO_4_ or ZnCl_2_, one of the main roles of the activating agent is to facilitate the dehydration of the carbonaceous macromolecular skeleton, which will influence the subsequent pyrolytic decomposition of the material, thereby reducing the production of tar and volatiles, and increasing the yield [23,24]. Likewise, the activating agent penetrates inside the channels of the botanical structure during the activation of the lignocellulosic material, causing them to swell, which will affect the generation of a more/less developed porous structure [11,23].

In the activation, intimate contact between the precursor and the activating agent is required. This confirms that the mixing procedure is important in the activation procedure. Thus, when using a solution of the activating agent, the precursor and the activating agent solution are mixed by stirring (impregnation) for several hours, up to 24 h, at temperatures ranging 60–150 °C depending on the chosen activating agent and precursor [9,10,11,12,18,23]. The solid activating agent can also be mixed with the precursor (physical mixing). This leads to remarkable porosity results, especially for alkaline hydroxides [10,12], being beneficial to avoid air contact as much as possible when using NaOH and KOH [10]. Since the activating (chemical) agent plays a major role in the porosity development, the weight ratio between activating agent and precursor (sometimes referred to as the degree of impregnation, especially when the activating agent is in solution) is a crucial factor determining the activation result [12]. Thus, the higher the degree of impregnation, the greater the specific surface area and the mean pore size developed in the AC [4,9,12,18], although it has a reverse influence on the final yield (defined as the ratio between the amount of AC prepared with respect to the amount of precursor used).

As stated, other important factors are the activation atmosphere, the activation temperature, and the time. Chemical activation is carried out under an inert atmosphere, usually passing a nitrogen gaseous stream through the mixture [12,13,18,19,25,37], although an oxidizing atmosphere can also be used [60,61,62]. Depending on the type of precursor and activating agent, activation can be carried out from 400 to 1000 °C, although usual temperatures range 450–750 °C (note that the required activation temperature is strongly linked to the activating agent) [4,9,12,18,37]. Activation temperature supposes an advantage over physical activation, which requires higher temperatures [3,9,12,25,37].

Another advantage of chemical activation, as a consequence of the chemical control in the precursors’ activation and the lower temperatures used, are the high yields generally achieved in chemical activation (for porosities similar to those obtained with physical activation) [4,9,63,64]. In contrast, a drawback for chemical activation is that a final washing step is required in order to eliminate the activating agent [9,11,13,25,37]. This can be carried out with distilled water (or with slightly acidified/basified water if hydroxides or acids are used as activating agents) [10,13]. It should be underlined that the washing effluent should be treated to recover the activating agent in an economical and environmentally sustainable process. Thus, chemical activation over lignocellulosic precursors is intriguing and frequently used due to the mild activation conditions and high yields generally obtained [4,9,11,13,18,25]. However, on an industrial scale, physical activation of lignocellulosic residues is extensively used since it is claimed to be cheaper, less polluting, and causes less furnace maintenance (corrosion) problems.

With the idea of depicting and summarizing the general trends on the activation yields and specific surface areas achieved by chemical activation, Figure 2 compiles some representative examples for lignocellulosic precursors. Figure 2A exemplifies the weight of precursor remaining as a function of the temperature used for activation (for one heating step activation procedures). In this figure, the larger the remaining weight (Y axis), the greater the final yield obtained. Figure 2A highlights that the AC yields reached depend on the precursor, the activation temperature, and the type of activating agent. Focusing on representative examples of chemical activation, and as indicated in Section 2.1, with coal precursors yields are generally higher than with lignocellulosic ones.

This figure also represents information about physical activation with CO_2_ of a lignocellulosic precursor, walnut shell. In this case, two heating steps in different atmospheres are required, pyrolysis in N_2_ to get the biochar (the auto-generated atmosphere during carbonization could/would be used in carbonization at an industrial scale), and heat-treatment in CO_2_ for the *proper* activation (see the corresponding temperature ranges depicted in the figure). CO_2_ activation implies larger temperatures and results in the lowest yield, mainly due to the important weight loss during pyrolysis (at 300–500 °C) in order to develop a (bio)char. If these data are compared with the activation of lignocellulosic materials impregnated with phosphoric acid or zinc chloride, it can be concluded that although initially (up to 200 °C) H_3_PO_4_ or ZnCl_2_ activation show higher weight loss, in the temperature range of 200–500 °C the weight loss is lower, and hence in chemical activation the yields are generally larger than for CO_2_ activation.

With the aim of enlightening the role of each activating agent, Figure 2B graphically represents the influence of the activation temperature on the relative porosity development (the combined information from Figure 2A,B provides an estimation of optimum activation temperature ranges for each chemical agent in order to simultaneously obtain a high porosity together with a high AC yield). Each activating agent shows an optimum temperature interval for the formation of porous structures and, although the plotted values are illustrative, and the porosities vary depending on the precursor and the selected experimental conditions (i.e., gas flow, heating rate, chemical agent/precursor ratio, …), the maximum surface areas for the shown examples are obtained for activation temperatures around 400 °C, 650 °C, 750 °C, and 880 °C for H_3_PO_4_, ZnCl_2_, KOH, and CO_2_ activation, respectively [4,9,11,25,37].

These notorious differences are a consequence of different physicochemical processes involved in the activation with each activating agent, see summary in Figure 2C. Thus, a literature review paying attention to the BET surface areas achieved by chemical activation provides values covering the 200–3000 m^2^/g range [4,9,11,18,25,37]. In relation with these values, it must be remarked that chemical activation could lead to ACs with BET surface areas higher than the theoretical limit value of 2630 m^2^/g [65].

In the case of physical activation, commented for comparison purposes, porosity is mainly produced via carbon gasification [13,25,37]. CO_2_ activation is an endothermic process that needs temperatures higher than 800 °C to be initiated. The process kinetics is controlled either by the rate of the chemical reaction or by diffusion, depending on the temperature range. At lower temperatures (i.e., below 920 °C), the rate of the chemical reaction (C + CO_2_ ⇌ 2 CO) is determinant, and CO_2_ molecules can penetrate the carbon structure before gasification [66]. As a result, a narrow pore size distribution is developed [66]. At higher temperatures, the process is mainly controlled by the diffusion rate of the reactants to the carbon surface. Gas molecules react at the outer part of the biochar particles, leading to some external particle burnout.

For chemical activation, some differences are observed depending on the chosen activating agent. In the case of potassium hydroxide activation, as an example, porosity is produced by two processes. Potassium hydroxide reacts with carbon, producing potassium carbonate, hydrogen, and some potassium compounds, namely metallic potassium and/or potassium oxide, and, as a result of such reaction, some carbon is consumed, generating porosity in the carbon-rich matrix [10,21,22]. Also, above temperatures around 650 °C, a catalyzed carbon gasification reaction by the CO_2_ evolved in the thermal decomposition of generated potassium carbonate takes place [10]. Simultaneously, a physical process enhancing porosity takes place at temperatures around 750 °C, consisting of the intercalation of the generated metallic potassium between graphene lamellas [67]. This expands the interlamellar graphene spacings, originating microporosity. If those activation temperatures are selected, i.e., 750–800 °C, “excessive” catalytic carbon gasification takes place, generating meso and microporosity [67].

In the case of H_3_PO_4_ and ZnCl_2_ activation, combined chemical and physical processes are also involved in the porosity generation [11,23]. During impregnation, both agents can penetrate the lignocellulosic macromolecular structure of the precursor, promoting the hydrolysis of the biopolymer components and swelling of the macromolecular structure [11,23]. H_3_PO_4_ and ZnCl_2_ promote the degradation of biopolymers during the heat treatment, accelerating the condensation and aromatization reactions. Thus, comparison between the weight loss in H_3_PO_4_ activation of wood with that in a similar heat-treatment of wood in absence of activating agent, shows in the former a high weight loss in the 100–200 °C temperature range (Figure 2A), whereas in the latter this weight loss usually occurs in the 250–400 °C temperature interval.

For H_3_PO_4_ and ZnCl_2_ activation, during the aromatization of biochars, some structural contraction takes place [11,23]. As a result, the reactants remain inside the developed macromolecular structure [11,23]. Hence, these activating agents act as template for porosity generation.

In the case of H_3_PO_4_, due to its acidity, chemical transformations occur at lower temperature than (weakly acidic or neutral) ZnCl_2_ [23]. For phosphoric acid activation, phosphate compounds inside the macromolecular lignocellulosic precursor structure transform into P_4_O_10_ from 400 °C, reacting with the carbonaceous material. Carbon gasification takes place, originating porosity, as well as widening any pristine porosity in the precursor. An adverse effect of the decomposition of phosphate linkages is the shrinkage of the macrostructure (at 350 °C), with some porosity reduction. In the case of ZnCl_2_, the shrinkage (with the same effects) takes place around 600 °C.

Thus, in general terms, Figure 2B exemplifies the different optimum activation temperature ranges for H_3_PO_4_ (350–500 °C), ZnCl_2_ (500–700 °C), and KOH (700–900 °C).

### 2.3. AC Structure

The result of the detailed processes involved in chemical activation is a material, activated carbon, whose structure could, in principle, be described as disorganized. Even today, research is being carried out to get to a correct interpretation of the AC structure, and even some authors prefer to describe these materials as disorganized nanoporous carbons [68]. In this sense, numerous models based on the idea that ACs are built from elementary structural units or blocks, of different shapes and sizes ranging from simple sheets of graphene, fullerene fragments, or quasi-graphitic fragments, have been developed to describe its structure [69,70,71]. The reason for such a variety of models is that they all give a fairly approximate view of the structure analyzed and due to the great variety of precursors, there is not a single type of structure that represents them all. In a very simplistic way, the AC structure can be described as an array of graphitic microcrystals (around 10 nm in size), made up of two to four defective graphene sheets which, in microdomains, are arranged in parallel in a sequence similar to that of graphite, although they lack a true crystallographic order in the direction perpendicular to them (they are curved, intertwined, and displaced) [65,69]. These microcrystals are randomly distributed, intertwining with each other either by weak van der Waals forces, especially in defective areas with unpaired electrons, or covalently, by aliphatic chain bridges. As a result of this very disorganized structure, AC is considered a “non-graphitizable” material: although it has a certain microcrystalline structure developed during its treatment at high temperatures (above 3000 °C), it does not extend to a macroscopic range [48,57]. Thus, as a result of this “disorder” and the greater interlamellar spacing in these materials (0.34–0.35 nm, compared to 0.335 nm in graphite), a high intrinsic porosity exists in an AC [65,69].

## 3. Further Properties (Different from Porosity) of the ACs Prepared by Chemical Activation That Merit to Be Analyzed

### 3.1. Surface Chemistry

As already commented, and as a result of their porous nanostructure, ACs are excellent adsorbents. An intensification of the weak physical interactions (van der Waals forces) between the surface (pores) of the carbon and the adsorbate molecules confined in these nanospaces occurs. Additionally, strong (chemical) interactions may appear between the activated carbon surface and the adsorbate molecules at specific sites, leading to strongly retained (chemisorbed) molecules [72]. Under some conditions the chemisorbed molecules even decompose, and, in this case, carbon plays a catalytic role. Surface groups and unsaturated atoms on the carbon surface are usually considered active sites.

The study of the surface chemistry in activated carbons can be carried out by a wide variety of experimental techniques and parameters [73]: chemical titration methods (Boehm and potentiometric) [74], measurement of carbon pH [75], point of zero charge and isoelectric point [76], spectroscopic methods (infrared spectroscopy—FTIR/DRIFTS, X-ray photoelectron spectroscopy—XPS/ESCA, K-edge X-ray near edge structure—XANES spectroscopy, electron spin resonance—ESR, and electron paramagnetic resonance—EPR) [77,78,79], temperature-programmed desorption—TPD [80] or calorimetric techniques [81]. These techniques are useful to determine the nature and quantity of the active sites present in an AC.

Within the structural heterogeneity of ACs, the active sites can roughly be located in two different sites: the basal planes of the polyaromatic sheets, which compose the micrographitic domains that constitute the structural framework of ACs, and the edges of these sheets. Structural (XRD) and microscopic (high-resolution transmission electron microscopy (HRTEM)) techniques provide information for such characterization. Henning highlighted, using TEM on graphite, the great difference in chemical reactivity of these two regions near lattice defects [82]. The higher chemical reactivity of carbon atoms present in the edges of the sheets (as well as in their defective areas) leads to strong bonds with other heteroatoms, causing the presence of carbon-oxygen, carbon-hydrogen, carbon-nitrogen, carbon-sulfur, and/or carbon-phosphorus surface compounds, among others, also known as surface groups or surface complexes [83].

The presence of heteroatoms is largely linked to the organic origin of the precursors (see Table 2 and Table 3). As previously indicated, during the heat treatment of the precursors, a large proportion of the heteroatoms is released as gases or tar. However, depending on the temperature reached, some remain within the chemical structure of the generated biochar [84]. Moreover, heteroatoms from the activating agents can also be incorporated into the carbonaceous structure during the activation process or chemical post-treatments [84]. Considering that oxygen usually represents 25–50 wt.% of the carbonaceous precursors (Table 2 and Table 3), it is not surprising that oxygen-containing groups are, by far, the most commonly present on the activated carbon surfaces. In addition, most activated agents are also oxygenated compounds, i.e., H_2_SO_4_, HNO_3_, H_3_PO_4_, KOH, NaOH. So, these agents can favor the generation of oxygen surface complexes during the activation process, being the surface properties of activated carbons mainly associated with the oxygen functionalities contents, the subject matter of a large number of investigations [85]. Figure 3 shows some representative surface groups on a carbon surface.

The chemical properties exhibited by activated carbons can be approached within the context of their Lewis acidic, basic, or neutral character [73,74,85]. On this basis, an acidic/basic character is presented by the different types of oxygen surface complexes. The type of oxygen surface complexes that confer acidic behavior on carbon surface has been well characterized, being related with carboxyls, lactones, lactols, phenols, hydroxyls, and anhydrides [73,74,82,85]. These oxygen functionalities provide hydrophilic sites on the hydrophobic carbon surface, improving its polar character. Concerning carbon basicity, several interpretations exist. One is related to the type of oxygen surface complexes, like carbonyls, ethers, quinones, chromene, pyrones, which contribute to the basicity of carbons [67]. Others, related with electron-rich Lewis basic sites, are mainly associated with regions of π electron density on the carbon basal planes, and, to a lesser extent, free-radical edge sites and dangling carbon atoms [78]. The basicity of carbons lowers the polarity of carbon surfaces and increases their hydrophobic character. In the case of ACs obtained by chemical activation, various studies have pointed out that the nature of their surface groups largely depends on the type of activating agent. Thus, literature states that activation with phosphoric acid generates acidic surface groups, those ACs prepared with ZnCl_2_ are slightly acidic or neutral, whereas KOH produces groups with high basicity [33,86,87,88]. An interesting example highlighting this is reported in the work of Luo et al., showing the reaction of ACs (prepared by H_3_PO_4_, ZnCl_2_, and KOH with the same activating agent concentrations) with acidic (NO_2_) or basic (NH_3_) molecules [88]. Their results conclude that basicity follows the order KOH > ZnCl_2_ > H_3_PO_4_, whereas for acidity it is H_3_PO_4_ > ZnCl_2_ > KOH. Note that the different functional groups are significantly affected by the impregnation conditions, i.e., activating agent concentration and soaking time [88]. However, these results are dependent on the conditions in which the activations are carried out, being the activation temperature ranges generally different, i.e., for H_3_PO_4_ (400–500 °C), ZnCl_2_ (500–700 °C), and KOH (700–900 °C). Focusing on the absolute content of surface oxygen groups, it strongly depends on the preparation conditions, varying from 100 to 8000 μmol/g AC [73,85,89].

**Figure 4:**
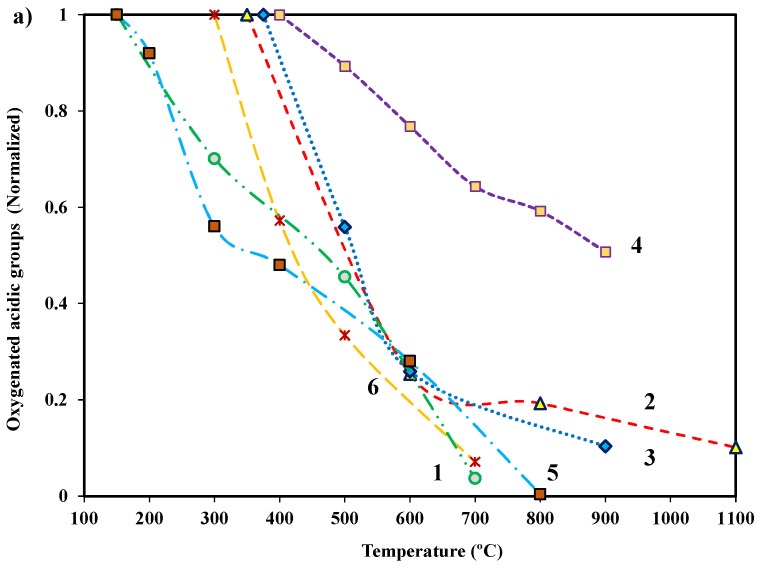

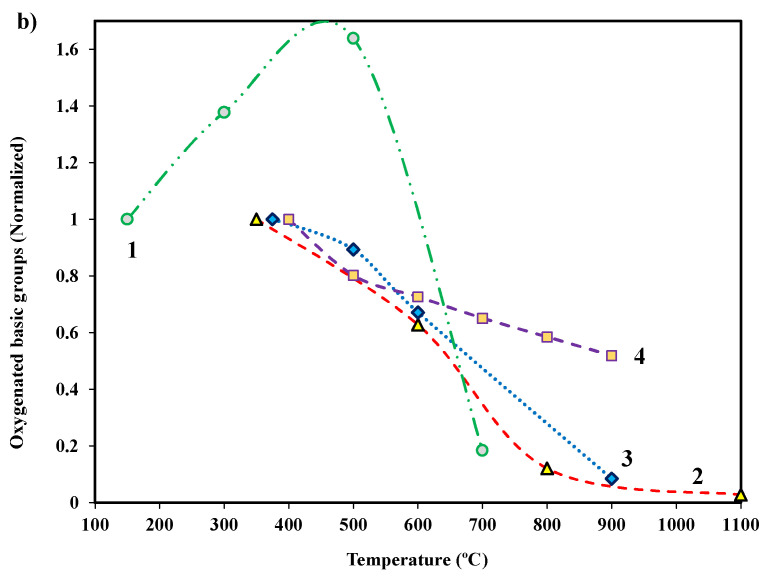
Evolution of the: (**a**) acidic and (**b**) basic oxygenated functional groups as the temperature of heat treatment increases for different series of activated carbons. Thermal treatments over an activated carbon prepared by chemical activation with KOH followed by ammonium peroxydisulfate oxidation, **series 1** [96]; an oxidized commercial AC, **series 2** [98]; an activated carbon prepared from olive stone by different acid treatments, **series 3** [45]; an AC prepared using potassium-rich wine stone precursor, **series 4** [91]; a commercial AC heat-treated and oxidized with two subsequent different treatments, **series 5** [99]; and a carbon oxidized with nitric acid and ammonium peroxydisulfate, **series 6** [100].

**Figure 1:**
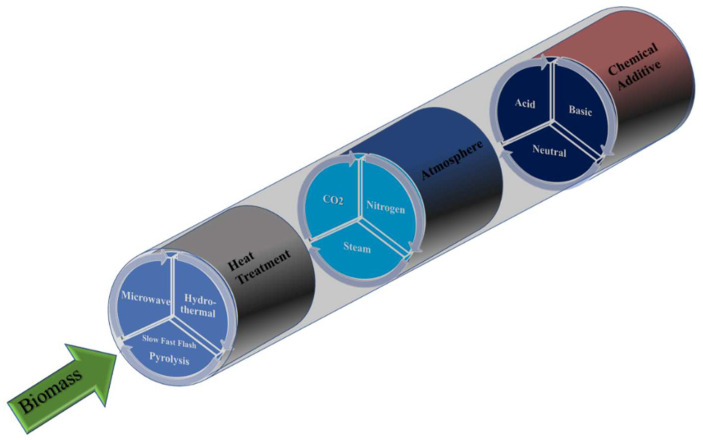
Experimental parameters involved in the preparation of ACs once the precursor has been selected.

**Figure 2:**
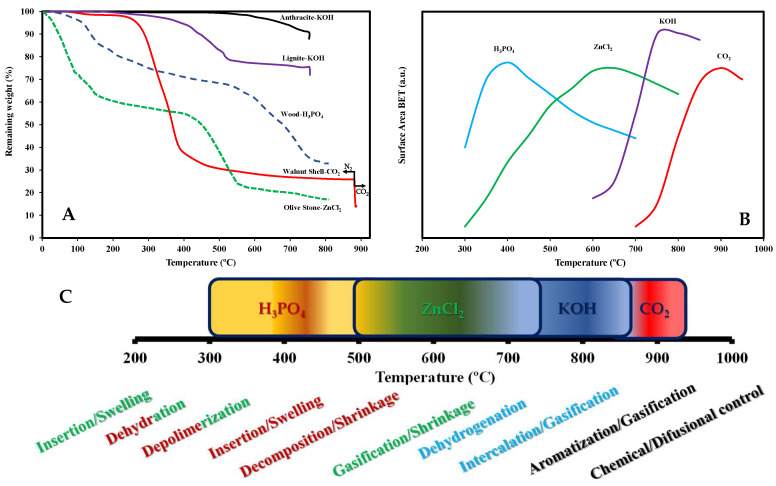
Illustrative figures to exemplify the influence of the experimental conditions used for chemical activation, namely activation temperature and chemical agent, on (**A**) the yield and (**B**) the surface area, using different chemical activating agents (and comparison with CO_2_ physical activation). (**C**) Range of temperatures used for the preparation of ACs by different chemical agents (and comparison with CO_2_ physical activation). The names in colors are relative to the physicochemical processes involved in the production of the lignocellulosic-derived ACs. Figure inspired from [9,11,17,18,19,22,23].

**Figure 3:**
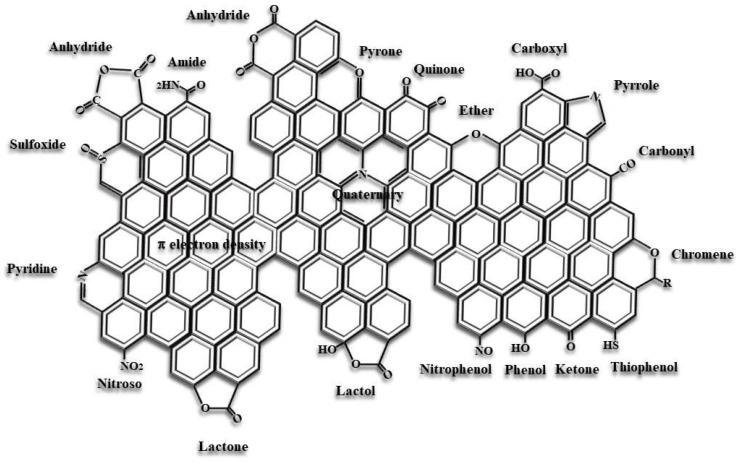
Surface groups on a carbon surface. Figure inspired by [73,74,77].

Nayak et al. has reported that the synthesis of activated carbons with ZnCl_2_ and KOH at the same activation temperature results in a similar content of surface groups [34]. This is a consequence of a well-known process of thermal decomposition of oxygenated functional groups as the temperature increases, which is related to their thermodynamic stability [90]. Thus, the surface chemistry generated by a fixed activating agent can be different depending on the heat treatment temperature. This is emphasized in Figure 4, which shows the evolution of the surface chemistry in a selection of ACs when using different heat treatment temperatures (note that in some cases the carbons had previously been oxidized).

As an example, Hasan Arslanoğlu has prepared activated carbons using potassium-rich wine stone precursor [91] and have reported a decrease in oxygenated groups (up to 50%), both acidic and basic ones, as treatment temperature increases from 400 to 900 °C (Figure 4, series 4). In their study, the contents of acidic surface groups double those of basic ones. In fact, a heat treatment in an inert atmosphere is a common method to increase the basicity of activated carbon surfaces as a consequence of the removal of acidic groups, which are those that decompose at lower temperatures [92,93,94].

All these examples have pointed out the importance and, in some cases, the difficulty of a correct assessment of surface chemistry in activated carbons, in general, and in biomass-derived ACs, in particular. Thus, if studies on the chemical activation of lignocellulosic precursors are taken into consideration, many of them perform a qualitative analysis (by FTIR) of such surface chemistry [18,19,25,32,89,95] and others some quantification. If quantifying, different techniques are used, including Boehm’s titration, XPS, TG, or temperature-programmed desorption [25,34,60,87,89,95,96], so the comparison between them is not easy/direct. Finally, zero charge potential is measured in some of these ACs as a direct indication of acidity/basicity of the carbon surface when being used in water applications [25,28,34,61,87,89], but results are variable (even very different for a common activating agent) depending on the preparation conditions of the AC. In this scenario, it is not so evident to assess which activating agent leads to larger surface groups generation. For example, it has been proved that chemical activation with phosphoric acid leads to the incorporation of phosphorus surface groups [97], although it has also been stated that the content in oxygen groups in an activated carbon can be even lower (or in the same range) as those in a thermally activated one. In contrast, a substantial surface oxygen groups’ content increase occurs via alkaline hydroxides’ activation [18,19].

A careful analysis of the surface chemistry characterization for “aged” (previously heat-treated) samples, those treated some time ago, has revealed changes with respect to the “fresh” heat-treated ones, which should be taken into account. These changes occur as a result of several processes. The first process was reported many years ago by Puri et al. [101], and more recently by Kyotani et al. [102] and Leon et al. [93]. When heat-treated ACs are exposed to the ambient atmosphere, oxygen chemisorption occurs, resulting in a decrease in the previously “reached” basicity. A second process, more recently reported by Maldonado-Hodar et al., has concluded that reorganization and mobility of oxygenated surface groups occur inside porosity after heat-treating ACs (see Figure 4b, series 1), and CO_2_-evolving groups are selectively transformed into CO-evolving groups [96]. It has been reported that micro-milling powdered activated carbon decreases adsorption capacity as a consequence of the formation of oxygen/hydrogen-containing functional groups incorporated from water during the milling step, causing ACs to become less hydrophobic [103]. All these facts indicate that if an activated carbon has been heat-treated in a certain manner so as to achieve the desired surface chemistry, the aging process modifies such surface chemistry leading, in general, to a decrease in the performance of the aged AC with respect to the initial one.

The surface chemistry of activated carbons can be modified, as previously stated, through various post-synthesis treatments [104,105], which do not only involve the acidic-basic character of the surfaces, but also the nature of the surface heteroatoms. As mentioned, the existence of certain oxygenated groups functionalities confers acidic properties, hence why the acidic character of ACs is frequently developed through heat treatment in the gas phase with oxygen, ozone, carbon dioxide, steam, and nitrous oxide (150–700 °C) [74,90,92], or liquid phase with oxidants such as nitric acid, hypochlorite, permanganate, or hydrogen peroxide [106,107,108].

Additionally, other functionalities and/or heteroatoms can be incorporated to increase the surface’s basic character through heat treatment in the gas phase using nitrogen-containing compounds via surface amination process, [109,110] or carbon-sulfur surface complexes by sulfuration processes [111,112]. The specificity towards the adsorption of certain compounds can be increased by anchoring coordinated ligands [113,114,115].

The mentioned methods have in common the use of a chemical compound and a thermal activation process to favor its interaction with the carbonaceous surface. In the last decade, other methods for the surface modification by activation of gaseous molecules have been analyzed, i.e., using microwaves [116], ultrasound [117,118,119], and non-thermal plasma treatments in different atmospheres, the last being one the most frequently employed [19]. Although the use of air is the most economical option, it develops a great variety of active species, both oxygenated, radical oxygen, ozone, and nitrogen oxides (NO, NO_2_, …), which make the control of the generated surface groups difficult [120]. In the case of air treatment, an increase in the amount of carbonyl and ester groups has been reported, and so has the basicity of activated carbon surface [121]. Contrarily, some authors have found that the use of O_2_-plasma (avoiding N_2_) increases the amount of (acid) carboxylic groups on the surface of AC [122]. Other authors have used NH_3_-plasma to modify the carbon surface [123], observing an increase in amino groups on the surface [123]. Recently, a mixture NH_3_/O_2_ –plasm has been used, resulting in an increase in acidic functional groups (–COOH) and basic groups (–NH_2_) [124].

In the case of lignocellulosic precursors, another approach has been tested in the last years to tailor the surface chemistry of ACs. Instead of the conventional pyrolysis used to obtain biochars, hydrothermal conversion of biomass has been an object of study, obtaining hydrochars [41,42]. These hydrochars are interesting precursors to produce activated carbon because their activation provides ACs with a higher density of oxygenated (acidic) functional groups than those obtained from “conventional” biochars [43,125,126].

### 3.2. Shape/Morphology

In materials’ production shaping is very important. For most fields, materials are optimally subjected to a shaping process to get the desired shape and size with the least loss in other properties, especially those of interest for each selected use. In the case of ACs, they can be conformed in different shapes depending on the application [127]: (i) Granulated activated carbon (GAC): this shape is usually obtained after the conventional activation of mineral or woody lignocellulosic-derived ACs. GACs are usually applied to the treatment of aqueous media due to their fast diffusion rate; (ii) Powdered activated carbon (PAC): in some cases, it is obtained after grinding and sieving AC particles whereas, in others, such as hydroxides activation, PAC is the activation product. They are directly used in processes that require rapid adsorption, for example, to clarify solutions; (iii) Activated carbon pellets (ACPs) and monoliths: they consist of AC pieces of fixed geometry (i.e., cylindrical, honeycomb, …), prepared from an intimate mixture of powdered activated carbon with a binding agent, which is well extruded obtaining pellets (particles of cylindrical symmetry) of diameter in the usual range 0.8–45 mm, or monoliths, pieces of greater dimensions formed using a hydraulic press for molding and compaction. Pellets and monoliths have high mechanical resistance (due to the binding agent used to maintain the AC particles together) which, together with their shape and low dust formation, makes them very suitable for the treatment of gas flows, given the low-pressure drop involved; (iv) Activated carbon spheres (SACs): they are usually prepared by activation of small spheres (size 0.35 to 0.80 mm) obtained from petroleum pitch molten in the presence of tetralin or naphthalene [128]. Recently, SACs have been obtained through the hydrothermal carbonization of pure cellulose [129], and starch [41,42,130]. The greatest advantage of SACs is their high packing density in comparison to the previous types of ACs, as a consequence of their spherical shape and reduced size, which allows more AC to be introduced per bed unit in adsorbent columns, together with their low-pressure drop; (v) Activated carbon fibers (ACFs): they are formed after the activation of carbon fibers; they present a fibrous structure with a very small fiber diameter (10–20 μm), which gives them high adsorption rate, as well as a minimum diffusion resistance [131]. An additional advantage is that they can be conformed into cloth or felts; (vi) Activated carbon nanotubes: they are obtained after the chemical activation of carbon nanotubes, whose main characteristic is being fibrous-shaped, with a diameter of just a few nanometers [39]; (vii) Ordered mesoporous activated carbons: in the form of an ordered structure on a nanometric scale, with a perfectly controlled mesoporosity size distribution. They are materials with meso and macroporosity volumes of up to 2 cm^3^g^−1^. They can be obtained by following two “opposite” procedures [132]: soft tempering and hard tempering.

Very innovative papers regarding shaped activated carbons materials have been published using phosphoric acid as activating agent, such as the activation of lignin-based fibers [97] or the use as precursors of hemp canes (with monolithic structure) from hemp stems [133].

Regardless their applications, the shapes and sizes of the ACs are of great importance from another operative point of view. ACs are used in continuous processes (treatment of gas or liquid flows), stirred processes (batch processes), or cyclic processes (such as PSA, pressure swing adsorption columns or TSA, temperature swing adsorption systems). After their use, a regeneration process of the AC is required involving, among others, cycles of downwards filtration and backwashing of the ACs, in the case of liquid applications, and thermal treatment with steam contraflow, or chemical regeneration, in gas applications [12,134,135,136]. Considering all these steps, ACs must have high mechanical strength and abrasion resistance properties to avoid operational problems, noting that their size could be reduced through abrasion and fragmentation during the application or regeneration steps [137,138].

### 3.3. Mechanical Properties

Attrition or hardness measures the mechanical strength, it determines carbon’s ability to withstand normal handling operations and serves as an important parameter for understanding the relative loss during the transportation, handling, use, and regeneration [14,23,40,67].

One of the parameters that strongly determines the mechanical properties of an AC is the type of precursor used. In this sense, to get good mechanical properties it seems reasonable to select a precursor with high mechanical strength. This has justified the use of hard coals (bituminous, anthracite) to get hard ACs, since some authors remark the low strength and low attrition resistance of biomass waste-derived ACs [12]. Paying attention to these important features, some authors indicate that precursors with a high lignin content (see Table 3) tend to have good mechanical properties [139]. Thus, shell precursor-based ACs show high hardness, being an example coconut-based ACs [140]. Also, wood precursor-based ACs can provide high mechanical strength [141], although it depends on the type of wood used [139]. Recently, Amoros-Pérez et al. have reported the preparation by physical activation with CO_2_ of spherical activated carbons (SACs) with high mechanical strength, taking advantage of the retention of the spherical shape by the hard natural seeds precursors, and compare them with those of three commercial ACs, one of them activated from wood with phosphoric acid [142]. The mechanical properties of their materials lie within the same order of magnitude, remarking the possibility of using lignocellulosic precursors [142]. In other work, AC monoliths have been prepared from cocoa husk [143]. In this study, the lignocellulosic nature of this precursor has shown to play an essential role in the preparation of the carbon monoliths [143]. However, the general impression is that this is one of the drawbacks to improve, since activation of biomass precursors with KOH leads to powder, with the consequent loss of mechanical properties, and activation with H_3_PO_4_ or ZnCl_2_, for example, derive in swelling of the precursor particles, with some loss in mechanical properties as well [11].

The presence of fatty matter and pectin in the composition of a biomass precursor gives it self-binding properties, allowing it to be shaped into discs (for improved mechanical properties, among others) without binding agents’ incorporation during compaction. Secondly, the lignocellulosic microstructure itself produces the formation of a carbon grid consisting of interwoven pleated laminae that persist during activation. This type of microstructure favors cohesion among the different microparticles of the carbonized material, endowing it with the appropriate mechanical properties [143].

Another parameter that favors an increase in mechanical resistance is the use of high carbonization (and activation) temperatures. In two-step heat treatment activation, the higher the carbonization temperature, the greater the condensation and growth of graphitic sheets, which leads to an increase in the mechanical resistance of the resulting material [57,58]. However, when focused on obtaining high-quality ACs although an “excessive” degree of condensation produces carbonized materials with high mechanical resistance, it is accompanied by two adverse aspects: the volume of pores is reduced, which is detrimental for many applications, and the reactivity of the carbonized materials (which should be further activated) decreases. This is an undesirable aspect and more vigorous conditions would be required to activate the char. Therefore, adequate control of the carbonization temperature is required to optimize these three parameters: porosity, reactivity, and mechanical resistance.

Regarding the influence of the type of activating agent used on the mechanical properties, its effect is less clear. P. Ravichandran et al. have paid attention to the influence of the activating agent and the preparation conditions (either one or two steps activation procedure) on hardness and attrition [144]. Their study has shown, for the three activating agents used, H_3_PO_4_, KOH, and ZnCl_2_, that higher hardness percentages are obtained in one step procedure [144]. With respect to the comparison between activating agents, they have shown that H_3_PO_4_ and KOH activation produces ACs with higher hardness than ZnCl_2_ [144]. Similar results have been reported by Khadiran et al. comparing H_3_PO_4_ and ZnCl_2_, obtaining such conclusions from the comparison between the crystallinity of the produced ACs [145]. In contrast, focusing on activated carbon monoliths prepared by chemical activation of olive stones, Nagakawa et al. have reported high mechanical strength both for phosphoric acid or zinc chloride [146].

In respect to attrition, P. Ravichandran et al. highlighted that attrition results are dependent on the carbon density, precursor materials, and activating agents used [14,23].

**Figure 5:**
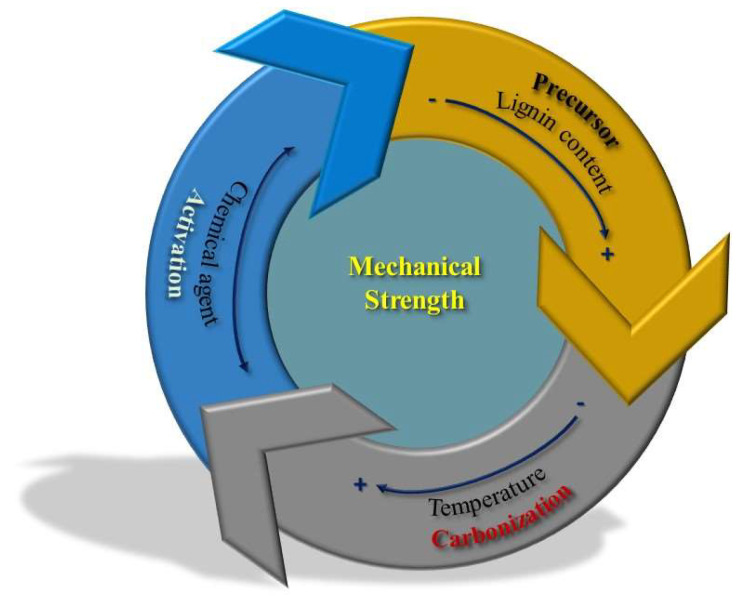
Parameters that influence the mechanical strength of prepared activated carbons. Note that although only carbonization temperature is cited, the activation temperature and other experimental parameters affect such properties.

Figure 5 summarizes some of the factors influencing the mechanical strength of chemically activated carbons, which usually range 3–60 MPa tensile strength and 60–95% attrition resistance [147,148].

Finally, another approach to obtain ACs with high mechanical properties is shaping activated carbons in a monolithic or pelletized form previously commented. This is usually carried out from a slurry of powdered activated carbon and a binder, which is compacted or extruded, and then carbonized and activated [127]. Many studies have been carried out with different binders, although most of them use coal-derived ACs as precursors. As an example, Lozano-Castelló et al. have studied the agglomeration of anthracite-derived ACs with several types of binders, concluding that most of the binders studied produce monoliths with good mechanical properties [149]. More recently, Ubago-Pérez et al. have worked on the preparation of granular and monolithic activated carbons from KOH-activation of olive stones using PVA as a binder [150]. In all cases, the adsorption capacities of the activated carbon monoliths were reduced with respect to the starting activated carbon due to the partial blocking of the porosity, which depends on the kind of binder used for the preparation of the monoliths [149]. A different approach in order to avoid such a decrease in textural properties is the synthesis of monolithic materials without binder [11,151]. As an example, Dolah et al. have obtained binderless electrode-activated carbon monoliths from mixtures of empty fruit bunch, KOH, and carbon nanotubes, which have been used as supercapacitors [151].

The published results highlight that the mechanical properties of ACs depend on the precursor, the temperature of heat treatment, the chemical agent and also the shape, and these all together determine the most suitable uses for any prepared material.

## 4. Aspects to Be Taken into Account for the Large-Scale Application of Chemical Activation for the Production of ACs

### 4.1. Scaling up of the Published Procedures and Economic Issues

To emphasize the importance of activated carbon’s world in economic terms it should be recalled that the AC market was valued at 3.2 million USD in 2021 and was expected to reach USD 4.5 million by 2028, with an estimated compound annual growth rate of 5.4% in forecast period 2021–2028 [152]. These numbers highlight its economic impact, not always analyzed in the literature. Thus, focusing on chemical activation of lignocellulosic precursors, most published studies deal with laboratory scale and only a limited number of papers have paid attention to the economic (and environmental) aspects involved in the large-scale production of activated carbons, being most of those papers related with the production of ACs for CO_2_ retention. As an example, Stavropoulos et al. have calculated the final costs of ACs prepared by chemical activation considering the raw material cost and the heating values [153]. They have shown that costs are very much dependent on the precursor, with the price of a chemically activated carbon derived from lignite around three times that for a wood-derived one, 4.22 versus 1.54 USD/Kg, respectively [153]. Asadi-Sangachini et al. have studied the chemical activation of walnut shells by phosphoric acid or potassium hydroxide, with the aim of developing low-cost highly efficient sorbents for CO_2_ capture [154]. These authors have proved that 2.5 H_3_PO_4_/precursor impregnation ratio is optimum for their application, leading to 1506 m^2^/g—essentially microporous derived sorbents, and have analyzed in detail the annual operating costs for the synthesis of AC by H_3_PO_4_ activation [154]. For such analysis, they have estimated 300 days/year production and two labor forces per shift (two shifts) for 24 h/day at USD11/h. Considering this, the estimated cost of AC production would be 1.83 USD/kg of eco-friendly AC [154]. Comparison of this cost with the values reported by Stavropoulos et al. points out that walnut shells can be considered as a cost-effective and promising biomass source for the preparation of ACs from the scale-up point of view, producing ACs with very good sorption properties.

Encouraged by the high cost of preparing solid adsorbents for CO_2_ post-combustion capture, Pramanik et al. have also studied in depth the cost of producing activated carbon by chemical activation (with KOH + alum, Al_2_(SO_4_)_3_·12H_2_O) by impregnation method [32]. A low-cost and abundantly available biomass precursor, non-fodder cotton stalk agro-residue, has been selected for this purpose [32]. In their study, which has conducted to the preparation of an activating carbon exhibiting a notable BET surface area (2695 m^2^/g), they accomplished a detailed techno-economic viability study of commercial-scale production, market size, costs, and revenues [32]. Considering some explained economic assumptions, they have concluded that 3.78 USD/kg would be the cost in a plant producing 2 tons/day of porous carbon (net present value of 0.7 million USD, internal rate of return of 39.15%, and payback time of 3.47 years) [32]. This cost is in the same range as those for physical activation of lignocellulosic precursors by air, steam, or carbon dioxide activation, also compiled in their study, although the porosity development achieved by Pramanik et al. is much superior [32].

In close relationship with economic (and environmental) concerns, Namazi et al. have studied the use of white liquor (containing NaOH) as an activating agent of pulp mill sludge, focusing on the influence of experimental conditions such as activation time and temperature, and NaOH/precursor ratio on the achieved surface areas and yields [155]. Their study is indeed very interesting for solving the issues associated with sludge disposal at pulp mills, applying an available activating agent source in chemical activation procedures. These authors have proved that their approach could improve the economy of the activated carbon production process [155]. However, a large amount of white liquor is needed for the production of activated carbon: 2 tons of dried white liquor are consumed to convert 1 ton of the dried pulp mill sludge into activated carbon, producing 500 kg of activated carbon [155]. In line with this study, Hasan Arslanoğlu has prepared activated carbons using potassium-rich wine stone precursor, proving the interest in such processes for economic (and environmental) reasons [91].

### 4.2. Environmental Concerns

Environmental concerns in chemical activation are indeed important, keeping in mind the toxicity of the chemical activating agents (i.e., ZnCl_2_ is not a desirable/preferred activating agent for those reasons) and any gaseous or liquid stream from the preparation procedure, and the possibility of recycling the chemical activating agent, strongly desirable. In this line, the investigation and use of mild conditions activation conditions is a tendency, with interesting approaches making use of different residues as carbon precursors [11,13,18,25].

After a detailed literature survey about the possibility of recycling the chemical activating agents, it can be concluded that the number of related studies is really scarce, and mainly devoted to KOH activation. This is probably because potassium hydroxide is one of the preferred activating agents from several points of view, including high porosity, very narrow micropore size distribution, and high final activated carbon yield [10].

Yuan et al. have tried to analyze if it is feasible to recover and reuse KOH in an activation process and, for that purpose, they have paid attention to the reaction mechanism and the prevalent reactions in two different temperature ranges, below and above 900 °C [156]. They have concluded that below 900 °C the dominant activation reaction follows this stoichiometry:

6KOH + 4C ⇌ K_2_CO_3_ + 4K + 3H_2_ + 3CO

Above 900 °C, K_2_CO_3_ is not stable and decomposes. Therefore, the prevalent activation reaction at this temperature range is:

KOH + C ⇌ CO + K + 0.5H_2_

In both cases, K can react with water, producing KOH and H_2_, which assures the activating agent recyclability [156]. As these authors have stated, unreacted KOH should also be taken into consideration [156].

Their study has aimed to conclude that ¾ of the potassium present in the activated product can be removed by water washing, and a majority of the potassium extracted with water is in the form of carbonate [156]. This potassium compound should be recovered and/or reused, due to environmental and economic concerns. Un-extracted potassium is likely metallic potassium intercalated in the carbon matrix [156].

Recently, Pramanik et al. proposed the recyclability of KOH during chemical activation by KOH + alum solution [32]. In their study, the supernatant solution containing dissolved KOH has been concentrated and recycled to the next cycle of impregnation [32]. These authors have paid attention to other interesting environmental issues, since their experimental system is thought to improve energy efficiency and they have accentuated the possibility of recovering a potassium-derived by-product to be used as fertilizer [32]. A very interesting approach, from an environmental point of view, has been presented by Nowrouzi et al., dealing with KOH activation of a water tissue in a process with zero liquid effluents discharge [95]. Recently, Głowniak et al. have paid attention to the preparation of activated carbons using a green precursor (i.e., tannic acid) and a cheap activating agent based on a mixture of NaCl and ZnCl_2_, synthesizing activated carbons with surface areas ranging 1190–3060 m^2^/g [157].

These studies highlight the interest in green and sustainable procedures. However, much more effort is required for optimizing the production of “green” and sustainable activated carbons by chemical activation.

## 5. Conclusions

The increase in the AC demand due to its extensive use in different applications, including water potabilization has led, especially in the last decade, to intensive research on the use of lignocellulosic precursors to produce activated carbons by chemical activation. In this review, the main features of biomass precursors and the physicochemical processes that take place during the preparation of ACs have been presented.

The lignin content has shown to be an interesting parameter, with a strong influence on the AC yield. The different physicochemical interactions between the activating agents and the lignocellulosic components responsible for the porosity generation have been revisited. Thus, whereas dehydration and insertion/swelling are the most important steps in the generation of porosity for H_3_PO_4_ and ZnCl_2_ activation, in KOH activation the controlled carbon consumption reaction and the intercalation of metallic potassium prevail.

Attention is paid not only to the optimal porosity development but to other key properties of the ACs produced, such as the generated surface chemistry using different chemical agents. The shapes, sizes, and mechanical properties of the ACs have also been commented on due to their importance in the uses of the ACs. This aspect is analyzed briefly, together with the mechanical properties of ACs.

Finally, some economic and environmental concerns related to chemical activation are studied because of their interest in a feasible and sustainable use of lignocellulosic precursors to produce chemically activated carbons.

## Figures and Tables

**Table 1:** Raw materials used in the preparation of activated carbons [13,25,27,37].

**Wastes from the Agri-Food Industry**	
Fruit pits	Olive, avocado, apricot, cherry, plum, date, mango, peach
Nut shells	Almond, hazelnut, coconut, walnut, pistachio
Soft shells	Avocado, pomegranate, orange, banana, yucca, corn, watermelon
Seeds	Orange, guava, palm, rapeseed
Seed husk	Rice, wheat, oat, peanut, coffee, cocoa
Processing paste	Flaxseed, vinegar must, apple pulp, oil, coffee
Fibers	Coconut, palm, banana, jute
**Wastes from the Agricultural and Wood Sector**	
Stems and leaves for pruning and harvesting	Cereal straw (wheat), sunflower, cotton, hemp, esparto, bamboo, cane bagasse, corn, tobacco, vine, kenaf, jute, tea
Wood	Tree bark and/or sawdust (eucalyptus, fir, pine, holm oak, olive, acacia, palm …)
**Industrial and Municipal Wastes**	
Waste materials from organic compounds	Plastics (PVC, PET), tires, paper, cardboard, wastes from the pulp industry, from the pickling of skins, textile industry.
Inorganic wastes	Sewage sludge, steel industry sludge.
**Fossil Fuels and their Wastes**	
Coal	Peat, lignite, subituminous, anthracite, fly ash, coal tar
Petroleum/oil	Pitch, coke

**Table 2:** Elemental analysis of coals [38].

Precursor	Ultimate Analysis (% *w*/*w*), dmmf * Basis				
	C	H	O	N	S
Peat	50–60	6.0–6.5	30–35	1.5	1.0
Lignite	65–70	5.0–5.5	22–26	1.0	1.5
Subbituminous	70–76	5.0	15–22	1.0	3.0
Bituminous	76–87	4.0–5.0	10–15	2.0	4.0
Anthracite	90–95	2.0–3.0	1–3	1.0	-

* dmmf: dry mineral matter free.

## Data Availability

Not applicable.
